# Editorial: Risk of dietary hazardous substances and impact on human microbiota: possible role in several dysbiosis phenotypes, volume II

**DOI:** 10.3389/fmicb.2023.1221169

**Published:** 2023-06-13

**Authors:** Els Van Pamel, Alicia Ruiz-Rodríguez, Ana Rivas, Margarita Aguilera

**Affiliations:** ^1^Flanders Research Institute for Agriculture, Fisheries and Food (ILVO), Ghent, Belgium; ^2^Department of Microbiology, Faculty of Pharmacy, University of Granada, Granada, Spain; ^3^Institute of Nutrition and Food Technology “José Mataix” (INYTA), Centre of Biomedical Research, University of Granada, Granada, Spain; ^4^Institut de Investigación Biosanitaria Ibs, Granada, Spain

**Keywords:** xenobiotics, microbiome, probiotics, dysbiosis, One Health

Cumulative dietary exposure to hazardous substances such as environmental agri-food xenobiotics, additives, nanoparticles or food contact materials [pesticides, polycyclic aromatic hydrocarbon (PHA), benzene derivatives, bisphenols, phthalates, parabens (Monteagudo et al., 2021)] have increased worldwide, especially during the three past decades in a continuous manner in industrialized areas (Ortiz et al., 2022). Under the One Health concept, integrated data from studies including, microbiome, multiple omics and exposome (as the prolonged exposure to these chemical substances) (Figure 1), seem to be a sound combined approach to elucidate intricate factors and health effects by which microbiota dysbiosis and inflammation, obesity, insulin resistance, metabolic syndrome, even infertility can be triggered (Gruszecka-Kosowska et al., 2022).

**Figure 1:**
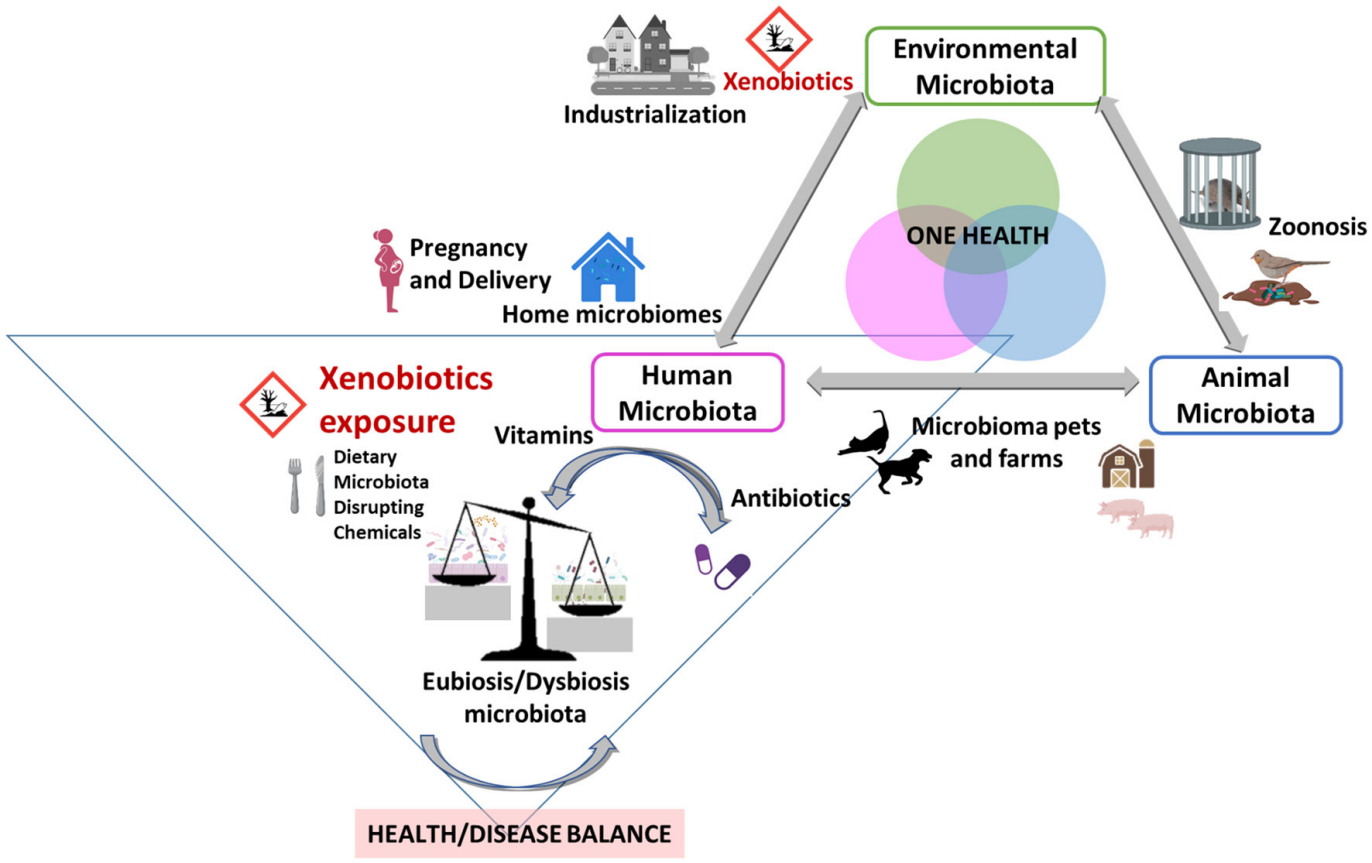
Risk of dietary hazardous substances or xenobiotic exposure and the impact on human microbiota under the One Health approach. Modified and adapted from Trinh et al. (2018).

Exposure to chemicals in early-life stages and during pregnancy can induce dysbiotic changes in the composition of the gut microbiome or alter the metabolic activity of the gut microbiota (Torres-Sánchez et al., 2023). Conversely, differences in the composition of the gut microbiota may alter the way in which environmental toxic substances are metabolized or even neutralized by biotics (probiotics, postbiotics, prebiotics, symbiotics). It starts to be understood that through families of biotools and enzymes such as azoreductases, nitroreductases, β-glucuronidases, sulfatases, and β-lyases, gut microorganisms can influence the metabolism of dietary xenobiotics. Moreover, the relevance of such interactions become an area of interest in toxicity and risk assessments, and the outcomes should be integrated in health policies and regulatory approaches.

The current Research Topic covers a collection of original research articles and reviews focusing on human microbiota dysbiosis, probiotics and next generation of probiotics for modulating associated pathophysiological phenotypes. In this sense, reviews agreed on the association of health disorders due to the impact of xenobiotics on the occurrence of microbiota dysbiosis.

Searching and demonstrating probiotic effects to equilibrate microbiota and health is a research common pursued activity. In addition to prolonged food preservation and improved organoleptic characteristics, nutritional and health-beneficial aspects have been linked to the consumption of fermented vegetables. Kumari et al. evaluated the probiotic and antidiabetic attributes of *Lactobacillaceae taxa* isolates from fermented beetroot. Six isolates categorized as *Lacticaseibacillus* spp. showed >95% similarity to *L. paracasei* and *L. casei* and were recognized as safe. Tolerance to bile salt and acid as well as to the gastrointestinal environment was observed. In addition, these isolates showed remarkable hydrophobicity abilities, autoaggregation and coaggregation capabilities, antibiotic and antimicrobial activities. Such properties indicate the notable probiotic potential of these six isolates. Compared to intact cells, the cell-free supernatant and cell-free extract exhibited a remarkably high inhibitory activity against the enzymes α-glucosidase and α-amylase of carbohydrate metabolism. As a result, the authors concluded that fermented beetroot harbors potential probiotic lactic acid bacteria with antidiabetic properties, and as such, their findings further support its use as fermented functional food.

Another research article on characteristics of strains isolated from fermented vegetable is the study of Chen et al.. These authors reported that *Bacillus amyloliquefaciens* WF2020 isolated from naturally fermented pickles may act as a potential probiotic, with synthesis of several active compounds, absence of virulence genes, susceptibility to various antibiotics, and the enhancement of *Caenorhabditis elegans* lifespan. This isolate was shown to be able to degrade aflatoxin B1 (AFB1) over a wide pH and temperature range. AFB1 degradation into metabolites with low or no mutagenicity and toxicity to *C. elegans* appeared to be mainly attributed to extracellular proteins or enzymes. Moreover, when co-incubated with *Aspergillus flavus, B. amyloliquefaciens* WF2020 affected its fungal growth and completely inhibited its AFB1 production. Additionally, the expression of ten aflatoxin pathway genes and two transcription factors (*alfR* and *alfS*) was suppressed. These findings suggest *B. amyloliquefaciens* WF2020 and/or its extracellular enzymes or proteins to be promising agents with potential application in view of protecting food and feeds from AFB1 contamination. Authors indicated however, that in order to elucidate the mechanisms of AFB1 degradation mediated by *B. amyloliquefaciens* WF2020, the purification of enzymes/proteins and the structure of degradation products warrants further investigation.

The research group of Ajeeb et al. explored the possible association between infant head-circumference-for-age-z-scores (HCAZ) during lactation (early vs. late) and possible shifts in the human milk microbiome. The findings of this cross-sectional study showed that in late lactation the HCAZ <-1 SD group (i.e., low or mild impairment in head-circumference) had more differentially abundant species of which several environmentally associated with soil, water, and animal sources and potentially opportunistic species belonging to the Actinobacteria phylum. In early lactation, the HCAZ ≥ −1 SD group showed more differentially abundant species with most of them being *Streptococcus* species from the Firmicutes phylum which are considered human colonizers associated with human milk. Although possible associations between brain growth of breastfed infants and milk microbiome during lactation are suggested by the authors, they also pinpoint out some limitations of this cross-sectional study and indicate the need for further investigating the cross talk between the human milk microbiome and the infant's brain. Last but not least, the authors suggested important potential roles of currently poorly characterized and unknown bacteria associated with head circumference, and as such, a substantial amount of knowledge on the role of human milk microbiome in infant growth and brain development yet to be discovered.

Another research paper concerning this matter is the one of Lopez Leyva et al. in which the impact of breastfeeding practices on the human-milk microbiome diversity and the differential abundance at the genera level is described. In this cross-sectional study, four clusters with distinct microbial communities segregating bacterial species by breastfeeding practices [i.e. exclusive (EBF) vs. non-exclusive (non-EBF)] and stage of lactation (i.e., early versus late stage) were identified. In EBF, bacterial composition shifted from the phyla Actinobacteria and Firmicutes in early lactation to a higher abundance of Bacteroidetes, Proteobacteria, and unknown bacteria in late lactation. For non-EBF (i.e., addition of *agüitas* (herbal teas) and/or complementary foods) a similar higher differential abundance of Actinobacteria and Firmicutes species in early lactation was observed, but in late lactation only Proteobacteria and not Bacteroidetes. While EBF is characterized by more differentially abundant bacteria, including commensal and lactic acid bacteria, for non-EBF the milk microbiome composition is modified by reducing the oral bacteria and introducing more environmentally sourced bacteria to the ecosystem. More general, the findings of the present study indicate that the stage of lactation is an important modifier of the human milk microbiome.

As is the case in Ajeeb et al. the article of Lopez Leyva et al. also described some limitations of the cross-sectional study as well as future needs, like addressing scientific gaps including uncovering possible associations of the human milk microbiome with sources of environmental bacteria, with functional properties and with early infant growth and development.

In their review, Ampatzoglou et al. innovatively highlighted three relevant areas of gut microbiome in the context of One Health: (i) the incorporation of the microbiome in food safety risk assessment of xenobiotics, (ii) the identification and application of beneficial microbial components to various areas under One Health, and (iii) specifically, in the context of antimicrobial resistance. For the area of risk assessment, the authors underline the challenging need to focus on the gut microbiota resilience, to look at function rather than composition, and to explore the active components of the gut microbiome. Such approach may contribute to establish specific biomarkers and is critical for advancing the incorporation of microbiome data in the risk assessment of xenobiotics. In addition, potential applications in various areas under One Health lie ahead seen that the human gut microbiome may be a promising source of beneficial components (microbes, enzymes, and bioactives) with the potential to metabolize xenobiotics. Possible areas of application are detoxification in animals or plants or biodegradation in the environment. This would be specifically of interest in view of ameliorating the global risk of antimicrobial resistance development. Finally, the authors emphasized that the concept of xenobiotic resistance development in the context of the gut microbiome may warrant further investigation.

Xenobiotics in the diet and the environment can also affect other microbiota encountered at body locations different from the gut. Within this respect, Feng et al. found short-chain inulin to modulate the cecal microbiota structure of leptin knockout mice receiving a high-fat diet. More specifically, it modulated the dysbiosis induced by high-fat diet, improved probiotics growth and inhibited conditioned pathogenic bacteria. Influences appeared to be significantly different in the wild-type and leptin knockout mice used in this study.

In summary, the articles of this Research Topic Volume II complement and enlarge previous contribution to the dysbiosis of microbiome following cumulative exposure to xenobiotics and contaminants as a step toward a better comprehension of this complex scientific health area.

## Author contributions

All authors listed have made a substantial, direct, and intellectual contribution to the work and approved it for publication.
